# Nurses’ perception of thirst in patients within palliative home care: a qualitative study

**DOI:** 10.1186/s12912-024-01985-y

**Published:** 2024-07-29

**Authors:** Caroline Lythell, Anne Söderlund Schaller, Tiny Jaarsma, Maria Friedrichsen

**Affiliations:** 1 Department of Health, Medicine and Caring Sciences, Linköping University, Linköping, Sweden; 2 Palliative Education and Research Centre, Vrinnevi Hospital, Norrköping, Sweden

**Keywords:** Palliative care, Thirst, Nurses, Content analysis

## Abstract

**Background:**

Thirst is the body’s natural urge to replenish fluids in response to a deficiency in hydration. Patients at the end of life gradually lose their independence and reach a point where they become unable to express their needs and can no longer drink on their own. In palliative care, the main advice is to provide regular oral care to relieve symptoms such as dry mouth and thirst. However, according to previous studies the prevalence of thirst and dry mouth remains.

**Aim:**

The aim of this study was to describe palliative care, nurses’ views and experiences of thirst in end-of-life care in specialist palliative care units.

**Methods:**

A qualitative interview study with an inductive approach was conducted. Eighteen nurses working in six different specialist palliative care units in different hospitals in Sweden were interviewed. The interviews were transcribed and analysed with a content analysis approach according to Graneheim and Lundman.

**Results:**

When nurses discuss thirst, they perceive thirst as a problem for the patient. This is attributable to various factors, including the patient breathing with an open mouth, a reduced level of awareness, and negligence on the part of the nursing staff. Signs of thirst are dry mouth, and frequently and intense sucking on the oral care stick during oral care. It also emerged that not all nurses perceived that dying patients experienced thirst. They believe that thirst is something that is reduced in the dying patient in the same way as hunger. The most important thing to them is to relieve the dry mouth by providing good oral care. Several issues, such as a lack of guidelines paired with the patient’s reduced consciousness and hence his/her lack of communication, make assessing thirst problematic.

**Conclusion:**

Nurses have different thoughts and experiences about thirst, where some perceive patients as thirsty while others perceive them as having a dry mouth. Nurses expressed that both evidence and guidelines are lacking.

## Introduction

In the World Health Organization (WHO) definition of palliative care, the relief of symptoms and other discomforts is essential [1]. This is also central to Kolcaba’s “Theory of comfort”. Kolcaba describes “Comfort” as an absence of discomfort, or a relief and well-being and should always be interpreted from the perspective of the patient. Assisting the patient to achieve well-being in terms of physical, psycho-spiritual, socio-cultural, and environmental aspects to reduce patient discomfort is the most important aspect of this theory. The theory states that well-being is a human need, especially for ill people so that they can achieve relief from their symptoms [2, 3].Thirst can be a distressing symptom for patients receiving palliative care [4, 5]. Nursing in palliative care aims to optimise the quality of life for both patients with serious illness, and their families, by treating and preventing suffering. This care includes the continuum of illnesses including the physical, psychosocial, emotional, and spiritual needs of seriously ill patients [6].

Thirst is the subjective sensation of a desire to drink something that cannot be ignored [7]. It is the body’s response to restore the fluid balance [8, 9]. In palliative care, patients facing severe illnesses are susceptible to experiencing thirst due to factors such as dehydration, imbalances in electrolytes, low blood pressure, and limited mobility, all of which can contribute to decreased oral intake [10, 11]. Patients in end-of-life care gradually lose their independence and become increasingly dependent on family and health care professionals (HCPs).

Ultimately, patients may reach a point where they are no longer able to drink independently, they may become unconscious, rendering them unable to articulate their needs or the discomfort they may be enduring. The current recommendation in the context of palliative care is to provide consistent oral care to alleviate symptoms such as dry mouth and thirst [12]. However, it is important to acknowledge that, despite this guidance, the prevalence of thirst and dry mouth is still common in palliative care settings [4, 13]. Some of the studies [4, 5, 13] that exist regarding the prevalence and severity of thirst are old, but no less relevant. In a study where thirst was investigated in patients with terminal cancer [5] it was found that 62% (*n* = 88) of the participants rated their thirst between 5 and 10 on a numeric scale ranging from 0 (“no feeling of thirst”) to 10 (“intolerable”). In another study where the aim was to investigate the relationship between symptoms and dehydration, it was found that 83% (*n* = 82) of the participants were thirsty [4].

Recently, several studies have been conducted on this subject from the experiences of other HCPs, such as physicians [14] and nurse’s aides [15] and also one study from the family member’s perspective [16]. However, nurses’ perceptions and views about thirst have not been studied before. Therefore, the aim of this study was to describe palliative care nurses’ experiences of thirst in end of life care in specialist palliative care units.

## Methods

### Design

This study was an inductive qualitative interview study with content analysis in accordance with Graneheim and Lundman [17]. The choice of this design was deliberate, aimed at providing a descriptive overview of individuals’ distinct experiences and to foster a slightly deeper comprehension of the phenomenon “thirst” among palliative care nurses.

### Sampling and setting

A purposeful sampling approach was used. Inclusion criteria for participation in the study stipulated that individuals must be employed as registered nurses in specialist palliative home care and possess a minimum of one year’s experience in caring for end-of-life patients.

The selection of informants was based on the following criteria: age, experience, gender and geographical distribution in order to achieve diversity in the sample. In total, the study involved the participation of 18 nurses.

Demographic data were gathered to ensure a representative geographical distribution, the study enlisted the participation of six distinct specialist palliative home care units located in various cities in south and south-east Sweden. These cities varied in population size, ranging from 3,000 to 1,000,000 residents. Palliative care nurses manage a wide range of activities within a holistic framework of understanding. This included the assessment and treatment of physical, emotional, social and spiritual health [18]. The role of the nurse is to assess and manage symptoms, communicate on different levels, and be a coordinator of care while using flexible and non-traditional methods in demanding situations, such as being present, to open oneself to the patient, to experience the patients’ lives, sensing their milieu and their history and context [19, 20].

### Data collection

Individual, semi-structured and voice-recorded interviews with nurses were conducted. An interview guide (see Table 1) was created by a research group [14] to capture the phenomenon. A pilot interview was conducted to test the understanding of the questions and the flow of questions. Clarifying and follow-up questions were used during the interviews, such as “Please, tell me more”; Please, explain; and How? and Why/Why not?

No changes were made after the pilot interview. Question number five was not used in this study. The interviews were conducted by A.S.S. (PhD in pain nursing) (*n* = 6), C.L. (PhD student in palliative nursing) (*n* = 2), U.R. (*n* = 2) (specialist nurse in palliative nursing), L.L. (*n* = 2) (specialist nurse in primary care) and E.J. (*n* = 6) (medical student). Fourteen of the interviews were performed in person, three of the interviews were conducted over the phone and one of the interviews was conducted over Skype. The interviews lasted between 8 and 62 min. Two professional transcribers and one medical student (E.J.) transcribed the interviews.

**Table 1:** Interview guide

1. Have you thought about whether end-of-life patients can be thirsty? If so, how?
2. Do you think end-of-life patients suffer from thirst? Justify!
3. Do you have a policy in your unit regarding thirst? Why/why not?
4. What are your colleagues’ views on thirst in patients at the end of life?
5. Can there be any ethical problems with patients who are thirsty?
6. Do you check whether a patient at the end of life is thirsty? How do you assess whether a patient is thirsty? If yes, what checks do you make? If not, why not?
7. Do you do anything to quench the patient’s thirst? What do you do? What do you think works best/worst?
8. Have you ever discussed thirst with relatives of patients at the end of life? Tell me!
9. How do you think one could work to quench thirst?
10. Is there anything I have not asked about thirst that you think is important to share?

### Data analysis

The analysis is based on Graneheim and Lundman’s manifest content analysis [17]. The interviews were read through by two authors (C.L. and M.F.) as a whole to get a sense of the content. After all interviews were read through, they were read again and this time meaning units were picked out by one author (C.L.). The meaning units were condensed into fewer words while retaining the meaning of the original text. The condensed meaning units were then given a code. The codes are based on the manifest content, close to the original text. The codes were sorted according to similarities and differences and from these, categories and sub-categories were created. All stages of the analysis were critiqued, reviewed by another author (M.F.), and revised. Finally, two more authors (A.S.S. and T.J) evaluated and reviewed the analysis procedure. No new data emerged after 18 interviews. Table 2 shows an example of the analysis process.

This study is about nurses’ experiences of thirst. The question about the ethical aspects of thirst in the interview guide led to a wide range of responses from the nurses, so we had to separate the ethical experiences from those that were only about thirst. Therefore, two manuscripts were written.

**Table 2:** Examples from the analysis of meaning units, condensed meaning units, codes, sub-categories, and categories

Meaning units	Condensed meaning units	Code	Subcategories	Categories
“Because it’s kind of gradual, you usually stop eating and drinking gradually.” I.7	Patients stop eating and drinking gradually	Thirst decreases	Thirst decreases at the end of life	Nurses fluctuating perception of thirst
“But I have often wondered how, when the patient can no longer communicate how much they suffer from thirst and dry mouth when they lie with their mouth open.” I.2	Wonder if patients suffer from thirst when they can no longer communicate.	Patients cannot communicate their thirst	Lack of communication	Difficulties assessing thirst
“No, we don’t have, no, we don’t have any guidelines (on thirst), except that we say it’s important, and we can go by ROAG*, and assess.” I.16	We don’t have any guidelines on thirst.	No guidelines on thirst.	Lack of guidelines	Difficulties in assessing thirst

*ROAG (Revised Oral Assessment Guide)

## Results

### Demographic data

Of the 18 participants, 14 were female and four were male. The mean age was 46 years. Two were masters in palliative nursing care. Table 3 lists the demographic data of the 18 participating nurses.

**Table 3:** Demographic data of the participating nurses

Gender	
Female/Male	14/4
Age (year)	
Mean (Median) Min-max	46 (45) 26–62
Working time	
Full time Part time	9 9
***Working experience in palliative care (years)***	
Mean (Median) Min-max	13.1 [8] 1–38
***Professional education***	
Bachelor in nursing	13
Masters in nursing (advanced level):	
*Anaesthesia* *Primary care* *Palliative care*	1 2 2

**Fig. 1:**
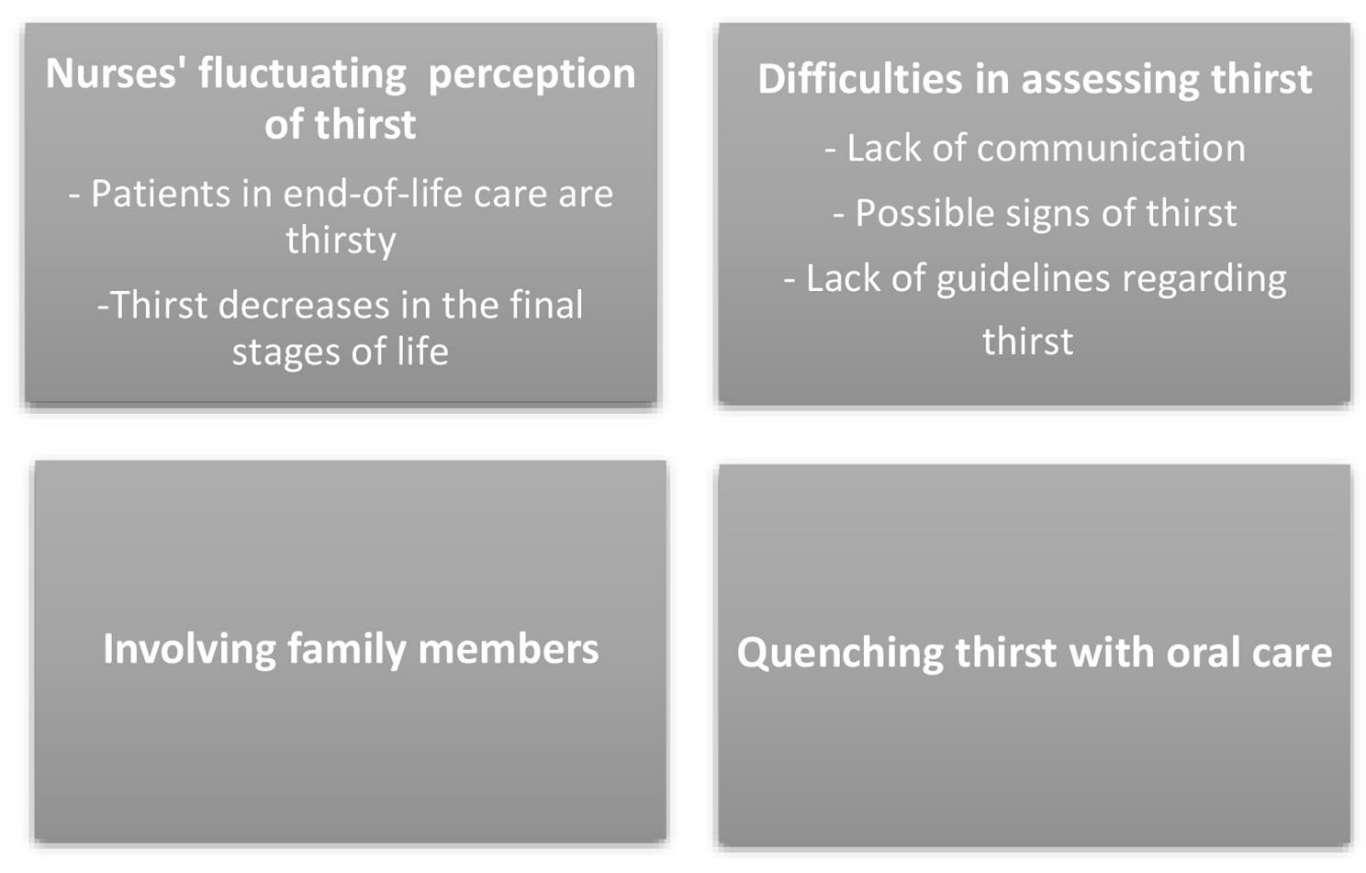
An overview of categories and sub-categories

The analysis resulted in four categories. The categories were: Nurses fluctuating perception of thirst; Difficulties in assessing thirst; Involving family members; Quenching thirst with oral care. Fig. 1 provides an overview of the categories.

### Nurses’ fluctuating perception of thirst

This category, Nurse’s fluctuating perception of thirst, contains the subcategories: Patient’s in end-of-life care are thirsty; and Thirst decreases in the final stages of life.

#### Patients in end-of-life care are thirsty

Some of the nurses had observed thirst in palliative care patients. They considered it likely that the sensation of thirst could arise, even if they thought the patient might not always be distressed by this. The nurses described that they had heard patients express thirst in their final days. The nurses reasoned that the patient’s dry mouth and reduced or non-existent fluid intake should lead to thirst in the patients. Furthermore, the nurses also indicated that the intensity of thirst varied depending on how close the patient was to the end of life. However, some nurses said that they and other healthcare professionals in palliative care usually told family members that the patient does not feel thirsty at the end of life, that hunger and thirst was something that subsides, despite their initial thoughts of patients actually being thirsty.

*“What we say is that the patient is not usually bothered by thirst at the end of life. You don’t get hungry, and you don’t get thirsty and things like that, so it’s very interesting, I think. Of course, we can occasionally sense that someone is bothered by thirst.” I.1*.

Some nurses described that the patients were thirsty but since that they no longer had the ability to swallow, they could not drink to quench their thirst.

*“There are those who are very thirsty but they have difficulty swallowing as well.” I.14*.

#### Thirst decreases in the final stages of life

Other nurses reported that they did not feel that thirst was present in palliative care at all. To them, thirst was considered an infrequent occurrence, and they attributed discomfort in the mouth to causes other than thirst.

*“Those [colleagues] I have spoken to also think that thirst is not so common. Instead, it is mainly this need to moisten the mouth.” I.9*.

Some of the nurses contended that patients receiving palliative care do not experience thirst, explaining that the natural process of dying diminishes the urge to drink, and patients are generally untroubled by thirst in their final stages of life. They described this reduced desire for fluids as a natural part of the dying process and suggested that attempting to provide fluids would only prolong the dying process and the patient’s suffering. While some nurses held firm convictions in this perspective, others were not as sure and they expressed they thought or believed this could be the case.

*“No, but it is natural anyway that all of this decreases, nutrition and fluids, and that it can rather be that you prolong a suffering.” 1.9*.

Several nurses mentioned that they did not believe the patient could feel thirst in the same way as they themselves could identify their own thirst. Despite their scepticism about patients suffering from thirst or requiring fluids in the final stages of life, they still acknowledged the potential need for some fluid, especially to moisten the mouth.

According to these nurses, as the need for nourishment and fluids decreased, the patient should be able to eat and drink to the extent that the patient wants and according to the patient’s own ability to provide for themselves. By that the nurses meant that they didn’t push the patient to drink or eat.

*“No, I think it’s [thirst] something that decreases. I think that you may feel a need to get some and especially to moisten your mouth and throat. But not that you feel thirsty like I myself can identify my own thirst.” 1.9*.

### Difficulties in assessing thirst

The category difficulty in assessing thirst consists of three subcategories: Lack of communication; Possible signs of thirst; and Lack of guidelines regarding thirst.

#### Lack of communication

Some nurses mentioned that it was difficult to know if the patient was thirsty as it was often impossible to communicate at the end of their life. The fact that the patient could not express their needs contributed to ignorance of the symptoms of thirst.

*“But we have certainly thought many times about how, when the patient can no longer communicate, how much they suffer from being thirsty, dry in the mouth when they lie with their mouth open.”* I.2.

Asking about thirst was something the nurses felt they needed to get better at. Some nurses described that they did not ask and one reason for not asking was that the patient was not conscious and could not answer. Some nurses pointed out that they may not have detected whether or not the patient was thirsty, as they did not actually actively ask about thirst.

The nurses described that from time to time the patient can actually communicate right up to the end. However, they also described that they were not used to asking about thirst, even if the patient was conscious and able to talk.

“*Yes, to ask about thirst, because we don’t really do that, that - we ask the patient about thirst as long as they can answer. Many times they can answer very close to the end by winking or in some way, we probably don’t do that. I don’t think we do that.” I.2*.

Questions about thirst were rather asked when the nurse could see signs that the patient was thirsty. The nurses said that they asked an open-ended general question about symptoms that bothered them, whereby thirst could come up as a symptom that concerned them.

*“Yes, but a clinical view, information from the patient of course. We often ask an overall general question about various symptoms and what is bothering the patient most right now, and then thirst can of course come up as a concern.” I.3*.

Some nurses shared that they did not perceive patients as thirsty, but questioned whether their experience might be influenced by the fact that they did not actively ask about the patients’ thirst.

*“I’ve been thinking about this very question, whether I feel that many people are thirsty, and I don’t think so! No! But that’s my experience, and I don’t know if it is because I should perhaps ask more actively as to whether they are thirsty?” I.13*.

The nurses wondered if it was really the loss of thirst, or if it was the loss of the patient’s initiative, resulting in the patient not being able to drink or describe their needs. When a patient had previously been very thirsty and could then no longer communicate, the nurses could not be sure that the patient’s thirst had disappeared. This meant that assessing thirst was difficult, since the signs of thirst decreased.

*“I’m not sure about that because, I mean that the thirst really decreases, or has their initiative decreased?” I.14*.

#### Possible signs of thirst

The nurses expressed that the patient expressed thirst in other ways than just by talking. The nurses explained that a patient could sometimes answer questions by blinking or nodding when they could no longer speak. The nurses said that it was mainly during oral care that thirst manifested itself. The nurses interpreted sucking on the oral care stick as a sign of thirst. Some patients, they said, grabbed the swab several times and sucked on it persistently to get fluids. The nurses expressed that they “thought” and “could imagine” that this was a sign of thirst, but that they could not know for sure that, this was the case.

*“I can imagine some of them, such as those who suck, when you have a swab with water on it and they really suck on it, then I think you are probably a bit thirsty.” I.4*.

However, other nurses said that they did not interpret the sucking of the oral care stick as a sign of thirst at all. They believed that when the patient sucked on the swab, it was a sucking reflex that was provoked by the oral care swab.

*“Yes, but it is, that you have read, that you have learned more from research and so on, that, that you have come to the conclusion that… it may not be… thirst that is experienced, but that it is more this reflexive.” 1.8*.

The nurses also had other ways to assess thirst when the patient could not express themselves. This was by looking at the degree of dry mouth. Several nurses believed that dry mouth could be seen as a sign of thirst. In addition, several nurses perceived that a sign of thirst was when the patient received oral care, when they observed that the patient experienced a sense of well-being and was clearly relieved.

*“Some people who are very close to the end don’t have the ability to tell me that they are thirsty, but then you look at the patient, and you can see that their mouth is dry.” I.6*.

The nurses found it difficult to distinguish between thirst and a dry mouth. They also expressed that it was difficult to determine whether dry mouth could mean thirst or whether they were only experiencing their mouth as being dry, as patients were often unable to express their symptoms in the final stages. The nurses suspected that dry mouth could be a factor leading to thirst. The nurses said that they have learned that it is the degree of dry mouth that affects thirst.

*“When they can’t express it themselves, you look for signs such as whether they have a dry mouth, or whether their mouth is very dry. We can’t tell you how they feel, whether they’re just dry in the mouth or whether they’re really thirsty and want to drink, so it’s difficult, but I suspect that they can… feel thirsty.” 1.5*.

The nurses supported their assumptions about the patients thirst by comparing with their own experiences of thirst, that they themselves became thirsty when breathing with an open mouth, and believed that this should also be the case for patients.

*“No, but it depends on which patient it is, I just think about how thirsty I can get if I don’t drink, [and] if I lie with my mouth open.” I.4*.

#### Lack of guidelines regarding thirst

The majority of nurses said that there were no guidelines or that they were unsure if they had any procedures for assessing thirst in patients at the end of life. However, a part of their job description was to check oral status. Several nurses expressed that they assessed thirst in connection with oral care or when ROAG (Revised Oral Assessment Guide) was done, which many performed routinely. They had no specific routine regarding thirst.

*“No, not specifically thirst [guideline] but we do ROAG regularly.”* I.15.

Even though some of the nurses said that they had guidelines regarding oral care, they did not know if they were followed. The guidelines that the nurses referred to were about how often oral care should be performed. The intervals varied from oral care being performed every five to ten minutes to every three hours.

*“[I am] actually unsure, as to whether we have a written routine, with, intervals, and so on. However, it’s usually … one, two, three hours. That’s the range in which you help with (oral care) for inpatients.”* I.14.

### Involving family members

The nurses described that family members could recognise that the patient was suffering from thirst, and that it was important to be there to listen and take in the information. The nurses believed that family members did not always need any action; they primarily wanted to inform the nurse about what they had observed.

*“The family member can raise the question (to us) that, now my relative, now he or she is bothered by a lot of thirst, but then it’s usually not so much about asking what action they should take, but more as information to us that he drinks a lot or so. I.3*.

Some nurses described that family members often communicated a concern about the patient being thirsty and sometimes wanted the patient to receive intravenous fluids (drip) to compensate for any lack of oral fluid intake. The nurses described that it was common for family members to be worried when the patient was no longer eating or drinking, that this would accelerate the dying process and they were also worried about whether the patient would feel thirsty by not drinking. Concerns about thirst, and thoughts about drips were a central part of the communication with members of the family.

*“Yes, it often comes up [the question of the drip] that you wonders as a family member, when the drip is not inserted, when the patient reduces the intake, perhaps stops eating, stops drinking and how, how it’s… experienced by the patient and they often wonder if that is what accelerates the process [of dying].” I.10*.

For the nurses, it was of great importance to inform the relatives and explain why a drip was not always an appropriate measure. They believed that well-founded information could help relatives to better understand the situation. Actively communication and discussion with family members was seen as a key element of their work alleviating their concerns about thirst.

*“It may be that the family are very worried that their, well, relative, is thirsty and not getting fluids and wants us to provide a drip to make it easier, and then it’s very important to explain to relatives that doing so may be risky, that they can’t take advantage of it and then we need to inform them about other ways we can help them.” I.5*.

The nurses said that they used to encourage family members to perform oral care. However, this was only if there were family members present who could help with oral care. Otherwise, the nurses said that oral care was done during the planned home visit, which was often once or twice a day. If the patient was in a ward, oral care was carried out more often. The nurses said that the guidelines they had were to inform about how often oral care should be performed and what equipment could be used for this.

*“Guideline no, that you check the mouth and that based on that, you take action with information about, if you leave oral care sticks and inform that you can buy, if it works with a saliva stimulant or if you use oil or water or Vichy water, so that it … it is a … policy that we check, follow up, treat fungus … and give advice.”* I.10.

### Quenching thirst with oral care

Some of the nurses emphasised the importance of effective and good oral care to relieve the patient’s thirst and dry mouth to prevent discomfort related to thirst. They underlined how crucial it is for HCPs to often moisten a patient’s mouth, particularly in cases when the patient is no longer able to communicate their needs their needs. By providing careful oral care, the patient could ingest a smaller amount of fluid. Nurses wanted to emphasise the importance of oral care in avoiding or alleviating the patient’s experience of thirst. They emphasised that oral care is the intervention that can be taken in the event of thirst when an intravenous drip is not an option due to the risks of pulmonary oedema. The nurses explained that moisturising the oral mucosa could prevent the feeling of thirst.

*“And it’s very important that we do it so that they get that help, and when we do oral care, they can also get some fluids.”* I.5.

The nurses described various approaches to achieving good oral care, including the use of moistened oral care sticks, ice cubes, saliva-stimulating tablets, oral care oil and other specialised oral care products. According to the nurses, these measures were considered effective in relieving patients’ thirst and improving their well-being.

*“That’s probably what we can do, we dip the oral care stick in liquid, juice or water…. or lemon water, you do it several times and then you use mouth spray.”* I.12.

The intervals at which nurses performed oral care varied. Some nurses described that oral care was performed every five to ten minutes, others talked about every 20 min and some explained that oral care was performed every hour. As much as every three hours could be acceptable to perform oral care according to the nurses.

*“But it is usually…one, two, three hours. In that range, that you help with it (oral care), on patients.” I.14*.

The nurses expressed that they themselves found it difficult to be thirsty and argued that it also must be difficult for patients at the end of life who cannot express their thirst. The nurses must then do their utmost to alleviate the patient’s discomfort.

*“It’s very difficult when you’re thirsty, you know yourself when it’s difficult, when you can’t drink and especially patients at the end of life when they can’t express it and then, as I see it, you need to do everything you can to help them with it.”* I.5.

## Discussion

This study contributes new knowledge about the phenomenon of thirst from the palliative care nurses’ perspective, and how they view this symptom. Nurses were divided as to whether thirst even existed at the end of life, with some expressing that thirst did not occur at all nor was it a troublesome symptom, while others believed that thirst was a real problem for patients.

The nurses who argued that thirst was not a symptom that the patients suffered from, support their assumptions with arguments, such as thirst decreases gradually with the dying process, that the body is gradually shutting down and that the patient themselves chooses to reduce fluids and nutrition at the end of life. However, it has been refuted that thirst does not occur at the end of life. For example, in a recently published study that examined the most effective methods to quench thirst in palliative care patients, it was found that all participating patients rated thirst and dry mouth above 5 on a numerical rating scale (NRS) [21]. The nurses who did not experience thirst as a bothersome symptom in the current study described what patients need at the end of life as oral care, to routinely moisturise the patient’s mouth, and that drinking is not necessary to quench thirst.

Nurses had a common standpoint that oral care was important at the end of life, but for different reasons, some considered it important to quench the patient’s thirst and others to keep the mouth moist and prevent discomfort from dry mouth. However, the nurses’ approach to oral care differed from guidelines. The time span nurses referred to for performing oral care varied from every 5 min to up to every 3 h. According to guidelines, oral care should be performed at least every 30 min [22]. However, there are no internationally recognised guidelines that are based on scientific evidence. Therefore, the existing guidelines are questionable [23]. When asked what they do to relieve thirst, the nurses replied that they did regular oral care and also that they used products such as saliva stimulants, oils and other products specially developed for oral care. However, these products do not quench thirst [24], which may suggest that nurses do not differentiate between thirst and dry mouth.

The results show that nurses perform oral care differently and that evidence-based guidelines are lacking in the workplace. Lack of evidence for oral care and oral health in palliative care, leads to carelessness or the neglect of oral care [23]. This was supported in another study that reported that healthcare professionals do not take patients’ oral problems seriously. Patients reported that nurses were poor at inspecting the mouth without offering help or symptom relief [21]. According to Kolcaba’s theory, “theory of comfort”, caring is described as the process of assessing the patient’s needs for comfort, developing and implementing good care and assessing the patient’s comfort after the intervention. Part of making assessments involves asking about distressing symptoms. It is important that the nurse asks the patient about their symptoms and tries to alleviate them so that the patient can achieve physical comfort [2].

A study investigating whether oral care helped to quench thirst in an intensive care unit found that oral care relieved the experience of thirst for only one hour. It was also noted that only a small proportion of patients participating in the study complained of thirst, although all of them experienced thirst. There was no correlation between the patient’s perceived thirst and the nurse’s objective assessment of the patient’s dry mouth. The study recognised the importance of nurses actively assessing and enquiring about a patient’s thirst, and not relying on an objective assessment [25]. This is relevant, as the results in this current study show that oral care could be performed up to every 3 h according to the nurses, and that some nurses believed that a patient’s dry mouth could be a sign of thirst. This also means that a moisturised mouth does not indicate that the patient is not thirsty. Therefore, it is important to actually ask about thirst during the assessment, as it was found that patients did not make the nurses aware of this themselves, since the nurses in the current study described that they did not actually ask about thirst.

Nurses who did not believe their patients were thirsty interpreted sucking on the swab as a reflex rather than a symptom of thirst, as other nurses interpreted the sucking on the swab. Nurses interpreted patient behaviour differently. However, many of the interviewed nurses expressed that they “thought”, “pondered” and “did not know”. This indicates a lack of awareness and an uncertainty about the issue and the need for clear evidence-based guidelines.

Nurses who believed that thirst was a common problem among dying patients also stated that they routinely informed the patient’s family that thirst did not occur and that it did not cause problems for the patient, because that was what they have learnt. The nurses informed the family members about this when family members expressed concern about the patient feeling thirsty. Which could be because of the lack of evidence and knowledge.

Several nurses mentioned that they used the oral health assessment tool ROAG [26] to assess thirst. However, there are no questions about thirst in this instrument, which means that in the worst case thirst is not assessed at all. This opens up the question as to whether there is a lack of knowledge among the nurses regarding thirst, or whether it is because the assessment tool is about oral health, which can be related to thirst? Thirst seems to be a knowledge that nurses do not reflect on in palliative care.

### Implications for policy and practice

It is important that the nurse can recognise thirst in the patient in palliative care, to reduce distress and increase the patient’s comfort. To enable the nurse to do this, it is necessary to raise awareness of thirst. Today, the ROAG is used as an oral health assessment tool, which does not measure thirst, only dry mouth. Creating an instrument that also measures thirst is important to ensure patient comfort.

Another important part is to educate staff in the subject of thirst; it is today something that seems to be relatively unconsidered and to be able to help the patient, the nurse in palliative care must have knowledge of this symptom.

### Strengths and limitations

This study explored nurses’ perceptions, experiences, and views of thirst in palliative care. Up until recently, not much has been published in this area. A strength of the study is that the questions asked gave participants the opportunity to express their perceptions, views and experiences freely [27]. Another methodological strength of the study is the number of participants, the study is based on 18 interviews and after these 18 interviews, no new data emerged.

To achieve dependability, we have made the process transparent by indicating the number of interviewees, the length of the interviews, how they were conducted and by citing specific interviews to presented evidence. Analytical transparency is ensured by showing how the authors interpret and analyse the data. To ensure confirmability, several authors were involved in the analysis process. To increase confirmability, we present citations to support the results of the study.

To achieve transferability the study is presented as precisely as possible for the readers and a purposive sampling were used. The data might be transferable to similar settings in other countries, however, this study was conducted only in Sweden and therefore it can only show the spectrum of nurses’ perception of thirst in this country and it is not obvious that it can be transferred and compared to other countries so the transferability can be questioned.

The interviews were informative. However, some of the interviews were rather short and their quality could therefore be questioned. Although, the subject of thirst in palliative care is rather unexplored, so deeper answers and reflection cannot be expected. One other possible weakness of the study could be that the interviews were conducted by three different interviewers, and that some of the interviews were conducted via telephone/Skype, which could mean that some of the information is lost, such as body language.

## Conclusions

To conclude, among nurses in specialist palliative care, thirst and oral care in general is neglected and disregarded and often no distinction is made between thirst and oral care. Oral care is a vital component of palliative care and should be a natural and prioritised part of the care, performed regularly and frequently, particularly if it should have any effect to relieve thirst. Oral health has a great impact on the patient’s overall health and is an important part of holistic care since it is always present and has a great impact on the patient’s well-being [28]. The “Theory of Comfort” can be helpful to the nurse as it emphasises the importance of providing comfort as an essential part of nursing care. It encourages nurses to focus on meeting the needs of their patients and to provide care that promotes comfort and well-being. Overall, the “Theory of Comfort” provides a framework for nurses to deliver high-quality care and improve the patient experience [18]. Healthcare professionals can provide better care and treatment to patients and improve their quality of life and experience by applying its principles to their care and treatment [2].

## Data Availability

Not applicable.
